# Failures in preclinical and clinical trials of c-Met inhibitors: evaluation of pathway activity as a promising selection criterion

**DOI:** 10.18632/oncotarget.26546

**Published:** 2019-01-04

**Authors:** Veronica S. Hughes, Dietmar W. Siemann

**Affiliations:** ^1^ University of Florida, Department of Radiation Oncology, UF Health Cancer Center, Gainesville, FL 32608, USA

**Keywords:** cancer, c-Met, small molecule inhibitor, microenvironment, HGF

## Abstract

C-Met is a frequently overexpressed or amplified receptor tyrosine kinase involved in metastatic-related functions, including migration, invasion, cell survival, and angiogenesis. Because of its role in cancer progression and metastasis, many inhibitors have been developed to target this pathway. Unfortunately, most c-Met inhibitor clinical trials have failed to show significant improvement in survival of cancer patients. In these trials tumor type, protein overexpression, or gene amplification are the primary selection criteria for patient inclusion. Our data show that none of these criteria are associated with c-Met pathway activation. Hence, it is conceivable that the majority of c-Met inhibitor clinical trial failures are the consequence of a lack of appropriate patient selection. Further complicating matters, c-Met inhibitors are routinely tested in preclinical studies in the presence of high levels of exogenous Hepatocyte Growth Factor (HGF), its activating ligand. In our studies, several tumor cell lines showed sensitivity to a c-Met inhibitor at high HGF concentrations (50 ng/mL). However, when the tumor lines were tested at HGF levels typically detected in human serum (0.4 to 0.8 ng/mL), inhibitor activity was lost. Thus testing c-Met inhibitors at non-physiological concentrations of HGF may lead to incorrect predictions of drug efficacy *in vivo*.

## INTRODUCTION

Cancer is one of the leading causes of death in the United States, with metastasis resulting in the majority of cancer-related deaths. Many oncoproteins contributing to tumor progression and metastasis have been pharmacologically targeted, with the goal of improving patient survival. Met was first identified in 1984 as an oncogene [1]. Since its discovery, it has been widely associated with tumor progression and poor prognosis in several types of malignancies, including prostate cancer, pulmonary adenocarcinoma, synovial sarcoma, melanoma, and ovarian carcinoma [2–6]. C-Met has also been linked to metastatic disease in prostate cancer [2].

C-Met is activated by the ligand Hepatocyte Growth Factor (HGF). The c-Met ligand HGF is normally secreted by stromal cells, including fibroblasts, osteocytes, astrocytes, and adipocytes [7–10]. Activation of the c-Met pathway results in the stimulation of downstream pathways involved in proliferation, scattering, migration, invasion, and survival, all factors important in the spread of tumor cells to distant locations. Angiogenesis is also influenced by the c-Met pathway and is an important step in the transition from a micrometastasis to a large, clinically detectable tumor. A small tumor (less than one cubic millimeter) can obtain oxygen through simple diffusion. To grow beyond this size, angiogenesis is induced to ensure oxygen delivery to the central parts of the tumor. C-Met plays an important role in this induction of angiogenesis.

Most commonly, dysregulation of the Met pathway occurs in the form of overexpression of c-Met and amplification of the gene. This has been noted in many tumor types, including breast, thyroid, colorectal, pancreatic, non-small cell lung, and prostate cancers [2, 11–15]. In a small percentage of patients, c-Met is constitutively activated by a mutation resulting in the deletion of exon 14. This occurs in 3–4% of non-small cell lung cancer (NSCLC) patients, and less frequently in other common forms of cancer [16]. The mutation results in a delayed degradation of phosphorylated c-Met, prolonging the signaling pathway. However, as mutation is quite rare, the majority of c-Met activation occurs through ligand-dependent mechanisms.

Because of the potential implications of c-Met on tumor progression and metastasis, there has been interest in interfering with this pathway as a therapeutic modality for cancer patients. A number of small molecule c-Met inhibitors have been developed and are currently in clinical trials. In our studies, we utilize BMS-777607, a small molecule ATP-competitive c-Met inhibitor evaluated in Phase I/II clinical trials in a variety of tumor types, including metastatic gastroesophageal cancer, hormone refractory prostate cancer, head and neck squamous cell carcinoma, and type 1 papillary renal cell carcinoma [17].

Preclinical studies of BMS-777607 and other c-Met inhibitors frequently employ the use of exogenous HGF to stimulate activation in the Met pathway. When studying the Met pathway *in vitro*, HGF is routinely added at concentrations of about 50 ng/mL. This produces robust phosphorylation of c-Met in many cell lines. However, serum HGF concentrations in healthy humans typically range from 0.3 to 0.5 ng/mL [18–20]. HGF concentrations can rise in patients with liver disease, myocardial infarction, and cancer [19–21]. For example, in patients with metastatic breast cancer, serum HGF concentrations of 0.8 ng/mL were measured [19].

In our studies, we examined the correlation between the commonly used biomarkers *MET* gene expression, total protein levels, and exon 14 mutation status, and how these markers related to activation status and sensitivity to c-Met inhibition. We explored the impact of exogenous HGF on sensitivity to c-Met inhibition in several cancer cell models. Finally, we determined the efficacy of c-Met inhibition in *in vivo* tumor models, using tumor models selected for either total protein expression or basal activation of the c-Met pathway.

While many tumor cell lines were insensitive to c-Met inhibition unless in the presence of high exogenous HGF, we identified a number of cell lines that were intrinsically sensitive to the inhibitor. Sensitivity to c-Met inhibition was associated with elevated basal activity of the c-Met pathway, which correlated with HGF secretion or deletion of exon 14.

## RESULTS

### Lack of correlation between mRNA expression, total protein expression, and activation of the c-Met protein across multiple tumor cell lines

**Figure 1:**
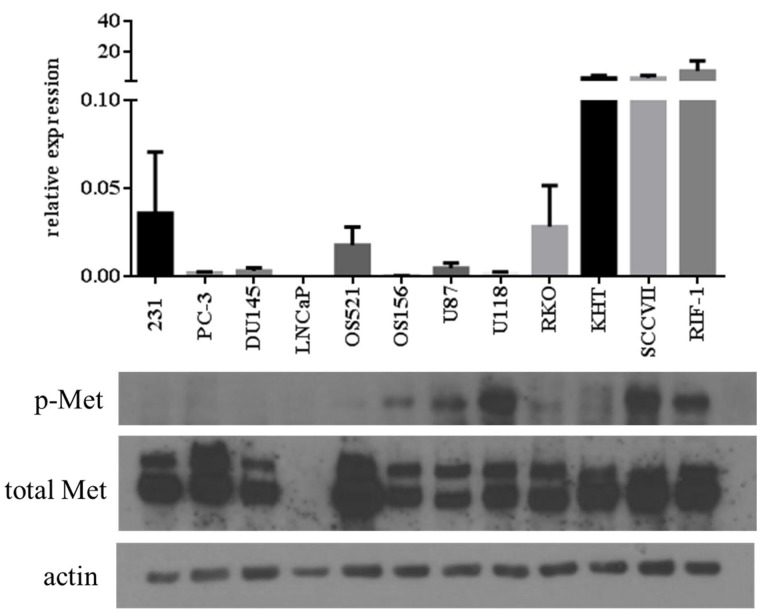
Correlation between MET RNA and protein expression levels across tumor cell lines: MDA-MB-231 (human breast), PC-3, DU145, LNCaP (human prostate), OS521, OS156 (human osteosarcoma), U87, U118 (human glioblastoma), RKO (human colorectal), KHT, RIF-1 (murine fibrosarcoma), and SCCVII (murine squamous cell carcinoma) Top: mRNA expression normalized to GAPDH. Bottom: Western blot of phospho-Met (Y1234/1235), total Met, and actin.

Twelve cell lines were compared for mRNA expression, total protein expression, and activation of the c-Met protein (Figure 1). Activation of c-Met was assessed using an antibody directed against phosphorylation site Y1234/1235. The results showed that there was no correlation between expression of mRNA and total c-Met protein. Most significantly, there was no relationship between either expression of mRNA or total c-Met protein, and phosphorylation of c-Met.

### Characterization of HGF secretion in cultured cells

**Figure 2:**
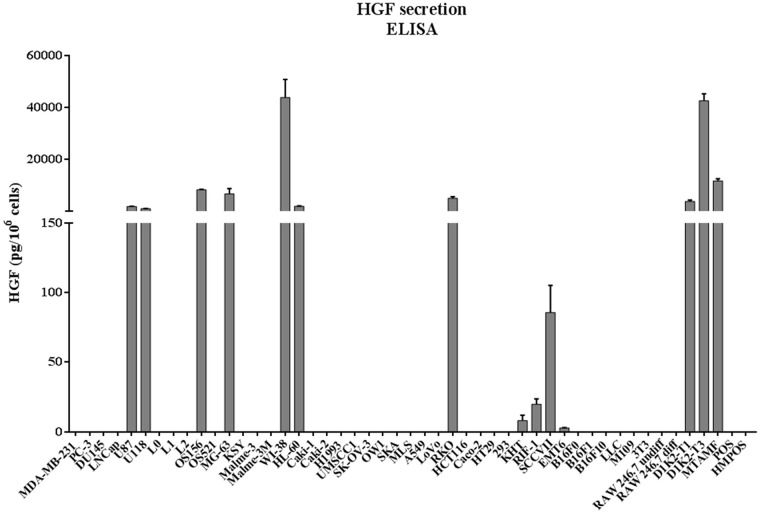
HGF secretion in conditioned media from cell culture Conditioned media was collected from cell culture after 24 hours, and analyzed for HGF concentration by ELISA. See Supplementary Materials for more information on cell lines.

Forty-nine cell lines were tested for HGF secretion. These included human, murine, and canine tumor cell lines, as well as human and murine stromal cells (Figure 2), with HGF normalized as pg/10^6^ cells. The following 14 cell lines were found to secrete HGF: U87, U118, OS156, MG-63, WI-38, HL-60, RKO, KHT, RIF-1, SCCVII, EMT6, D1K2-T1, D1K2-T3, and MTAMF.

### HGF secretion is associated with phosphorylation of Met

**Figure 3:**
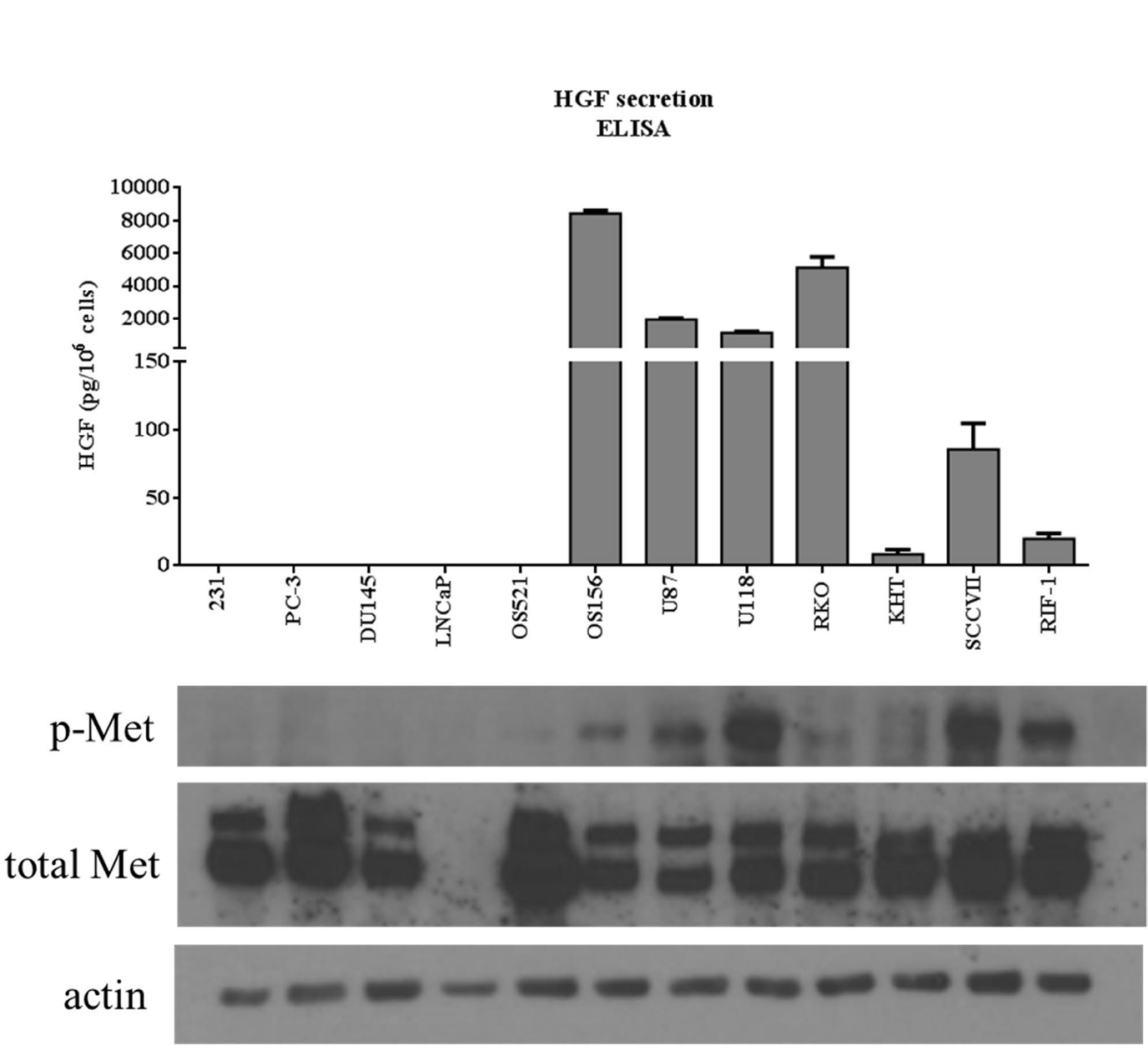
Comparison between HGF secretion and phosphorylation of c-Met Top: HGF secretion assessed by ELISA analysis of conditioned media. Data shown are selected tumor cell lines from Figure 2. Bottom: comparison of phosphorylation of c-Met (Y1234/1235) and total Met between tumor cell lines. Actin was used as a loading control. This western blot is a reprint of Figure 1.

A select number of non-HGF-secreting and HGF-secreting cells were selected from those analyzed in Figure 2: MDA-MB-231, PC-3, DU145, LNCaP, OS521, OS156, U87, U118, RKO, KHT, SCCVII, and RIF-1. These cell lines were chosen to represent both HGF-secreting and non-HGF-secreting cell lines. These particular cell lines were chosen as the lab has well-established *in vivo* metastasis models of these cell lines. HGF secretion profile was juxtaposed against the western blot originally presented in Figure 1. There is an association between HGF secretion and phosphorylation of c-Met (Figure 3) that is not seen when comparing total c-Met protein expression and pathway activity. Tumor cells that both secrete HGF and express c-Met are referred to in this paper as autocrine-activated tumor cells. Tumor cells that express c-Met but do not secrete HGF are referred to as paracrine-activated cell lines.

### Exon 14 mutation status in murine carcinoma cell lines

**Figure 4:**
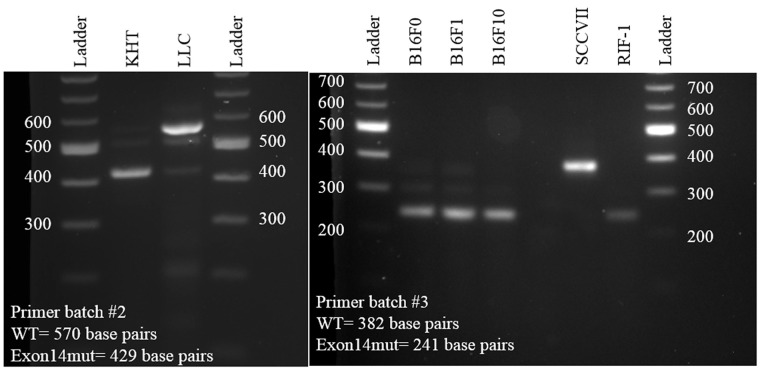
Determination of c-Met exon 14 mutation status for the three murine cell lines examined in Figure 3 (KHT, SCCVII, and RIF-1) LLC, B16F0, B16F1, and B16F10 were used as controls.

Several murine carcinoma cell lines were tested for deletion of exon 14, a mutation known to cause activation of the c-Met pathway. The SCCVII cell line was found to be wild type, while the KHT and RIF-1 cell lines both lack exon 14 (Figure 4).

### Inhibition of migration after treatment with BMS-777607 is dependent on HGF concentration in paracrine-activated tumor cells

**Figure 5:**
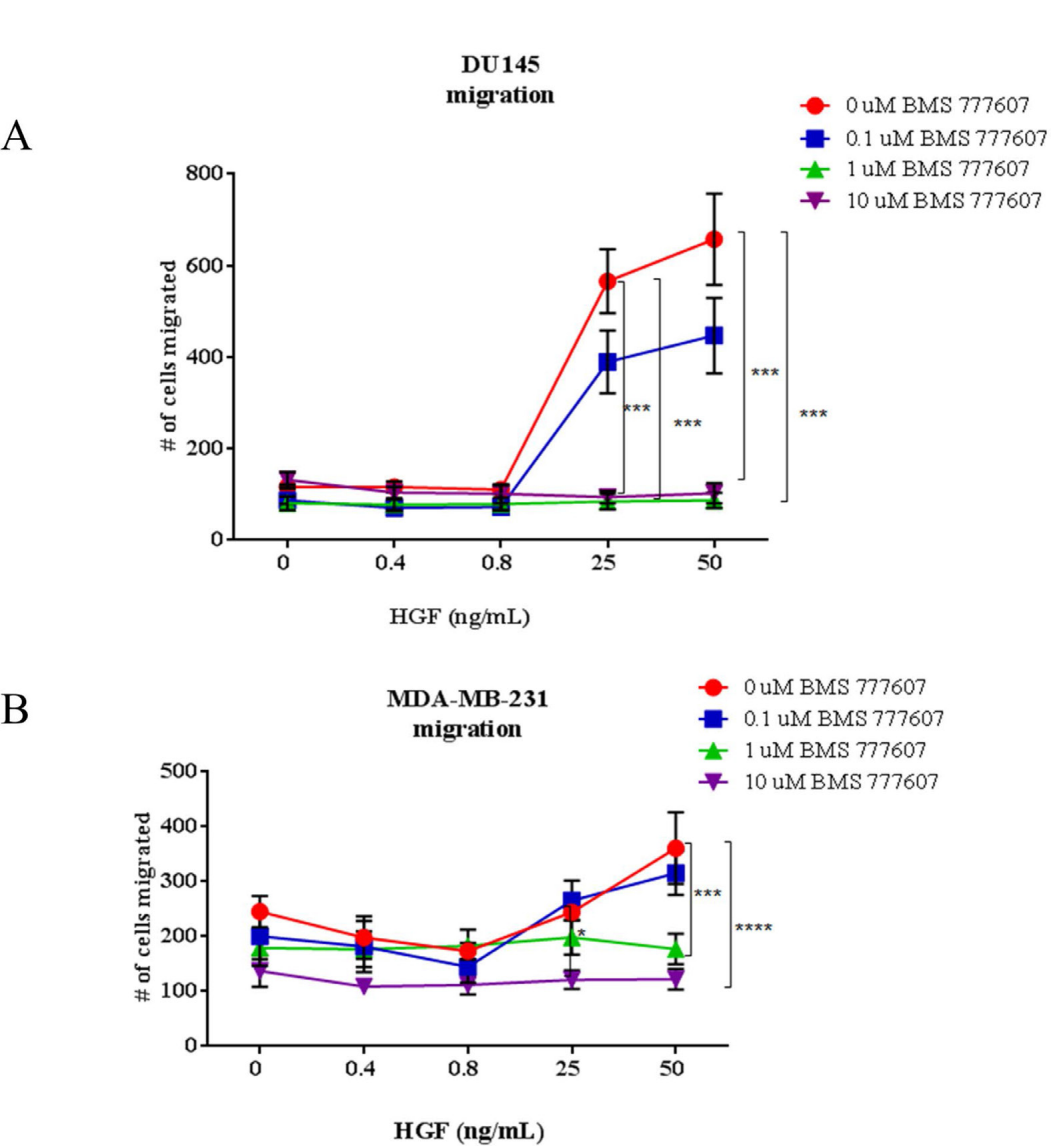
Transwell migration assays of paracrine-activated tumor cells (**A**) DU145 and (**B**) MDA-MB-231 migration assays. Cells were treated with BMS-777607 and/or HGF and allowed to migrate for 24 hours through an 8 μm pore membrane. Results are a combination of three independent experiments. Multiple comparisons ANOVA was performed using GraphPad Prism 6.0, with *p* < 0.05 considered significant. Mean with SEM bars are shown.

The human prostate cancer cell line DU145 and the human breast cancer cell line MDA-MB-231 were chosen to represent tumor cell lines with robust expression of total c-Met that did not secrete HGF; thus defined as paracrine-activated cell lines. Cells were assessed for their ability to migrate in the presence of BMS-777607 at low and high HGF concentrations (Figure 5). HGF was added at 0.4 and 0.8 ng/mL, to represent HGF concentrations found in the serum of a healthy human, and a patient with metastatic disease, respectively. HGF was also added at 25 and 50 ng/mL, to represent concentrations of HGF often used in literature. The c-Met inhibitor BMS-77607 was used at 0, 0.1, 1, or 10 μM. At 0.4 and 0.8 ng/mL HGF, the DU145 and the MDA-MB-231 cell lines did not demonstrate a significant inhibition of migration in the presence of BMS-777607. However, when 25 or 50 ng/mL HGF was present, migration of the cell lines was significantly impaired by the c-Met inhibitor.

### Autocrine-activated tumor cells are intrinsically sensitive to inhibition of migration by c-Met inhibition

**Figure 6:**
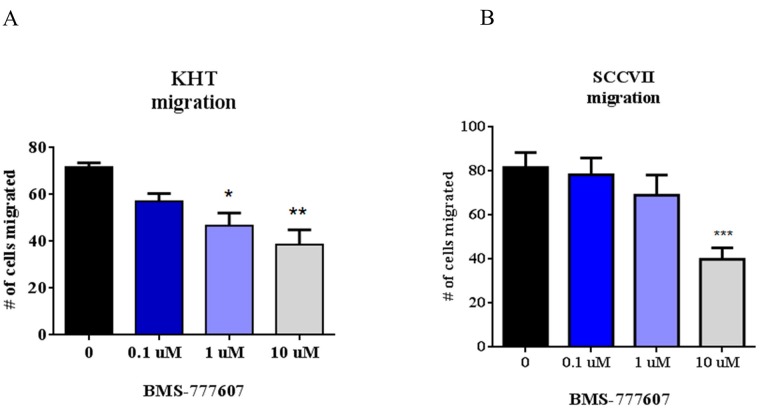
Transwell migration assays of autocrine-activated tumor cell lines (**A**) KHT and (**B**) SCCVII migration assays. Cells were treated with BMS-777607 and allowed to migrate for 24 hours through an 8 μm pore membrane. Results are a combination of three independent experiments. One way ANOVA was performed using GraphPad Prism 6.0, with *p* < 0.05 considered significant. Mean with SEM bars are shown.

While the paracrine-activated tumor cell lines showed a response to the c-Met inhibitor only in the presence of high levels of HGF, autocrine-activated tumor cell lines were intrinsically sensitive to the drug. Both the KHT and SCCVII squamous cell carcinoma secrete HGF and have elevated basal phosphorylation of Met, and as such, were predicted to be sensitive to c-Met inhibition. These cells were assessed for the ability to migrate in the presence of BMS-777607 (Figure 6). In contrast to the experiment presented in Figure 5, no exogenous HGF was added to the experimental conditions. Both cell lines demonstrated a statistically significant reduction in migration and invasion after treatment with the c-Met inhibitor.

### Inhibition of phospho-Met with BMS-777607 is dependent on HGF concentration in paracrine-activated tumor cells

**Figure 7:**
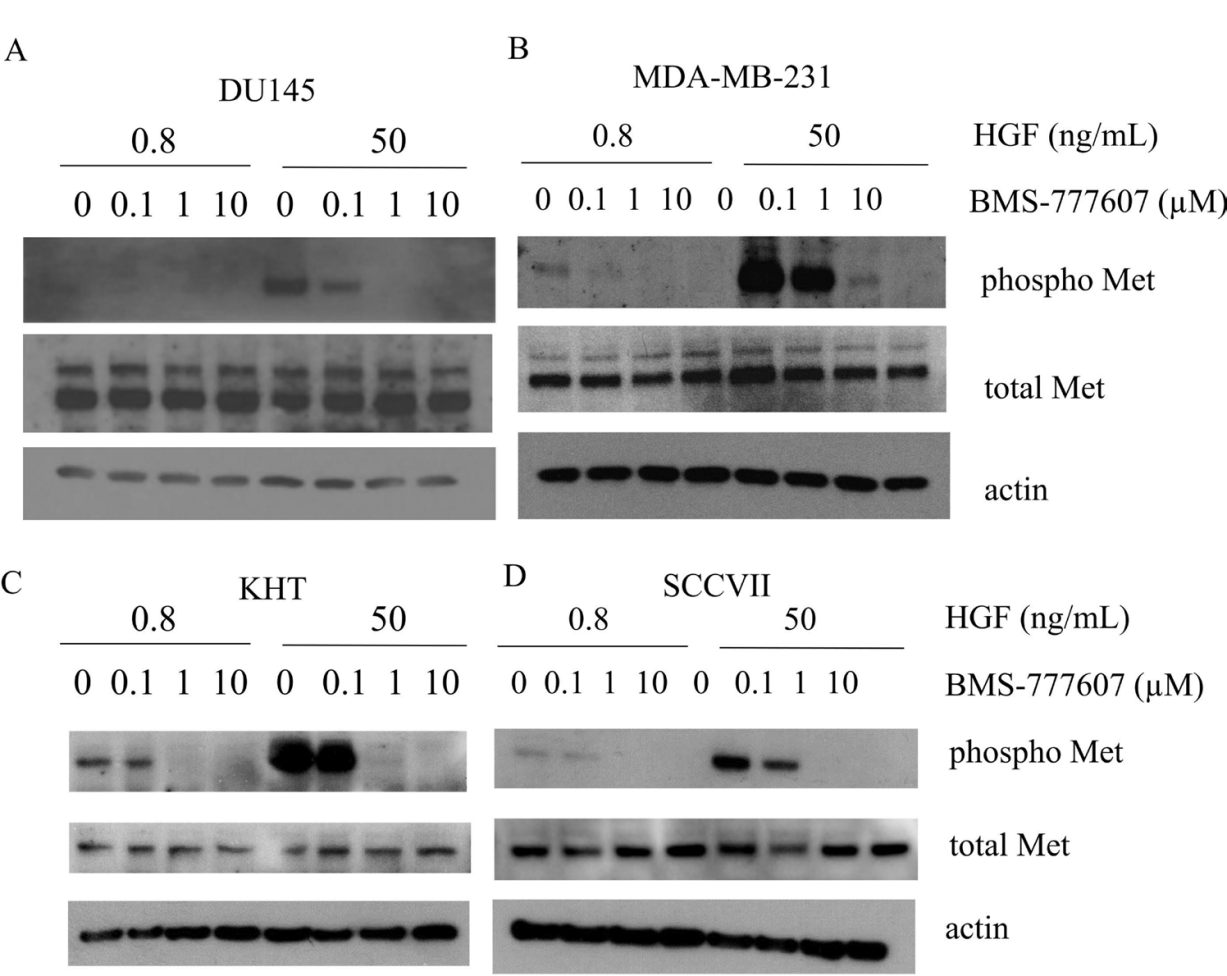
Phosphorylation of c-Met (Y1234/1235) after a 24 hour treatment with BMS-777607 (0, 0.1, 1, or 10 μM) and a 15 minute treatment with HGF (0.8 or 50 ng/mL) in (**A**) DU145, (**B**) MDA-MB-23, (**C**) KHT, and (**D**) SCCVII tumor cell lines. Actin is used as a loading control.

BMS-777607 was assessed for its ability to inhibit phosphorylation of Met in paracrine-activated cell lines in the presence of two concentrations of HGF: 0.8 and 50 ng/mL. As before, these concentrations of HGF were chosen to represent those found in the serum of a patient with metastatic disease, and the concentration typically used in literature, respectively. BMS-777607 was used at 0, 0.1, 1, or 10 μM. Cells were treated with BMS-777607 24 hours prior to harvesting the lysates, and HGF was added 15 minutes prior to harvest. DU145 and MDA-MB-231 cells treated with 0.8 ng/mL HGF showed no decrease in Met phosphorylation in response to BMS-777607 treatment. However, both cell lines showed a strong inhibition of phospho-Met when treated with BMS-777607 in the presence of 50 ng/mL HGF (Figure 7A, 7B).

### Inhibition of phospho-Met with BMS-777607 occurs without the need for exogenous HGF in autocrine-activated tumor cell lines

The KHT and SCCVII tumor cell lines were chosen to represent autocrine-activated cell lines. Both cell lines showed an appreciable decrease in phosphorylation of Met both at low and high concentrations of HGF (Figure 7C, 7D). Both cell lines also showed a robust phosphorylation increase in the presence of 50 ng/mL HGF.

### Tumor cell-induced angiogenesis is not inhibited by treatment with BMS-777607 in paracrine-activated cell lines

**Figure 8:**
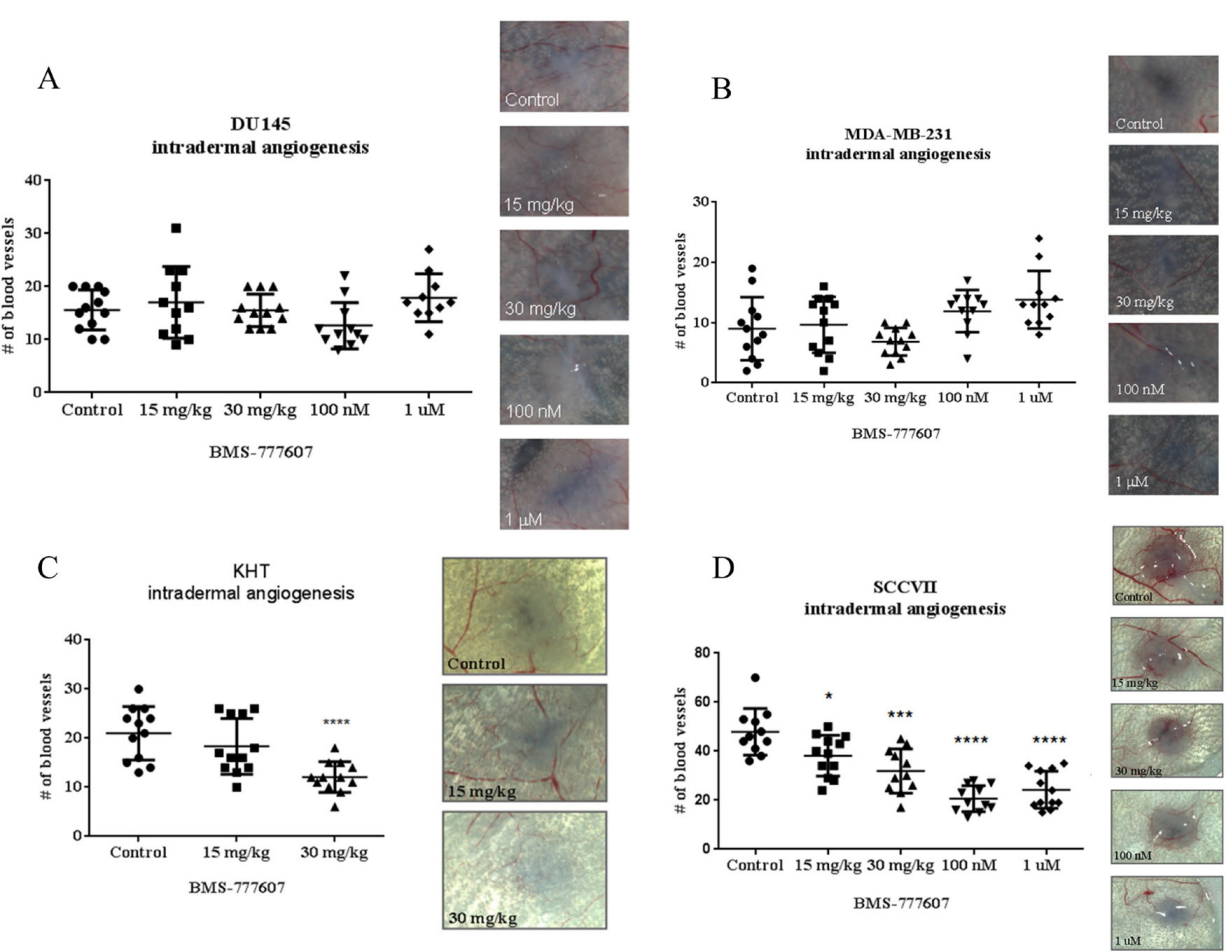
Intradermal angiogenesis assay (**A**) DU145, (**B**) MDA-MB-231, (**C**) KHT or (**D**) SCCVII tumor cells were injected intradermally into nude mice. Mice either received 15 or 30 mg/kg BMS-777607 daily for the duration of the experiment, or were injected with cells that had been pretreated for 24 hours with 100 nM or 1 μM of BMS-777607. Mann–Whitney *U* test was performed using GraphPad Prism 6.0, with *p* < 0.05 considered significant. Mean and SEM are shown. Representative images of tumor nodules are included.

Tumor cell induced-angiogenesis was measured using an intradermal angiogenesis model [22]. In this model, mice were inoculated with (i) BMS-777607 pretreated (100 nM or 1 μM for 24 hours) DU145 or MDA-MB-231 tumor cells or (ii) untreated tumor cells and given daily doses of BMS-777607 (15 or 30 mg/kg) by oral gavage. Neither pretreating the tumor cells nor treating the tumor cell bearing mice with BMS 777607 resulted in a reduction in the tumor cell initiated angiogenesis. (Figure 8A, 8B).

### *In vivo* tumor cell-induced angiogenesis is significantly impaired after c-Met inhibition in tumor models with basal activation of the c-Met pathway

The KHT and SCCVII tumor cell lines were assessed for the ability to induce angiogenesis *in vivo* (Figure 8C, 8D). Mice were injected intradermally with tumor cells and then received BMS-777607 daily for 3 days. In the case of the SCCVII tumor cell line, some mice were injected with tumor cells that were pretreated with 100 nM or 1 μM BMS-777607 for 24 hours prior to injection. Treatment with the c-Met inhibitor, whether via oral administration or by pretreatment of the tumor cells, significantly reduced angiogenesis in both tumor models.

### Experimental lung metastases of paracrine-activated tumor cells are not inhibited by treatment with BMS-777607

**Figure 9:**
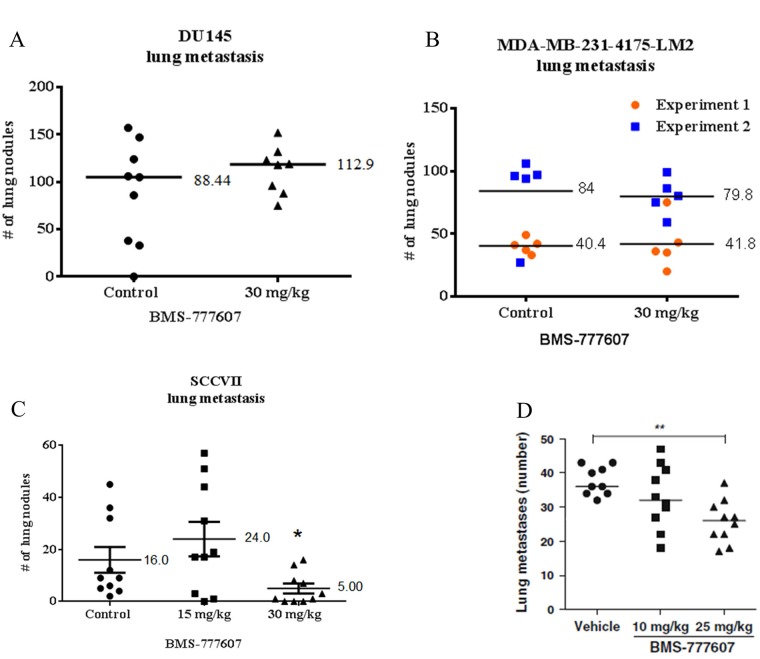
Lung metastasis assay (**A**) DU145 and (**B**) MDA-MB-231-4175-LM2 tumor cells were injected via tail vein into male and female NSG mice, respectively. Mice were treated with vehicle or 30 mg/kg BMS-777607 for 5 days following tumor injection. Lung nodules were allowed to grow for 7 or 6 weeks (DU145 or MDA-MB-231-4715-LM2, respectively). (**C**) SCCVII and (**D**) KHT tumor cells were injected via tail vein into female C3H/HeJ mice. Mice were treated with vehicle, 15 mg/kg, or 30 mg/kg BMS-777607 for 5 days following tumor injection. Lung nodules were allowed to grow for 3 weeks. (Panel **D** reprinted with permission). Following euthanasia, lungs were placed in Bouin's solution for 24 hours, and lung nodules were counted using a stereoscope. Mann–Whitney *U* test was performed using GraphPad Prism 6.0, with *p* < 0.05 considered significant. Mean and SEM are shown. The MDA-MB-231-4175-LM2 is a combination of two separate experiments, each group with an *n* = 5.

Experimental lung metastasis was assessed using a tail vein injection assay in NSG mice (Figure 9A, 9B). Mice were intravenously injected with tumor cells and treated with the c-Met inhibitor BMS-777607 for 5 days following tumor cell injection. Seven weeks after DU145 tumor cell injection, mice were euthanized. Metastasis formation of the DU145 tumor cell line was not reduced by treatment with the c-Met inhibitor BMS-777607. Similarly, BMS-777607 did not reduce lung metastasis formation in two independent experiments using the MDA-MB-231-4715-LM2 cell line.

### C-Met inhibition is effective in reducing lung metastases in autocrine-activated tumor cell lines

Previously published work from our lab demonstrated efficacy of the BMS-777607 c-Met inhibitor in reducing experimental lung metastasis in the KHT tumor cell line [23] (Figure 9D). In order to determine if the same effect was seen in the SCCVII cell line, mice were injected via tail vein with SCCVII cells and treated with BMS-777607 (Figure 9C). Mice receiving 30 mg/kg BMS-777607 developed significantly fewer lung SCCVII metastases than mice receiving vehicle.

### Correlation between phosphorylation and total protein expression varies between oncoproteins

Previously, phospho-Met was found to not correlate with total protein expression. An examination of several other cancer-related proteins revealed varying correlations between activation and total protein. In the case of the Src oncoprotein, phosphorylation and total protein levels were closely correlated. For the Akt protein, most samples also appeared to have correlation between phospho- and total protein. However, Axl, Fak, and Pax showed great disparity between activation status and total protein. For the proteins examined in this study, total protein expression was not a reliable indicator of phosphorylation status.

## DISCUSSION

C-Met inhibitors have been extensively examined in preclinical studies, where they were found to be well tolerated and effective as anti-cancer therapeutics. Based on these preclinical studies, several agents entered human trials. By and large these have performed poorly in clinical trials, and as a result, there has been diminishing interest in further pursuing this class of inhibitor. However, the authors contend that the assessment of c-Met inhibitors, in both preclinical and clinical evaluations, suffer from significant shortcomings.

**Figure 10:**
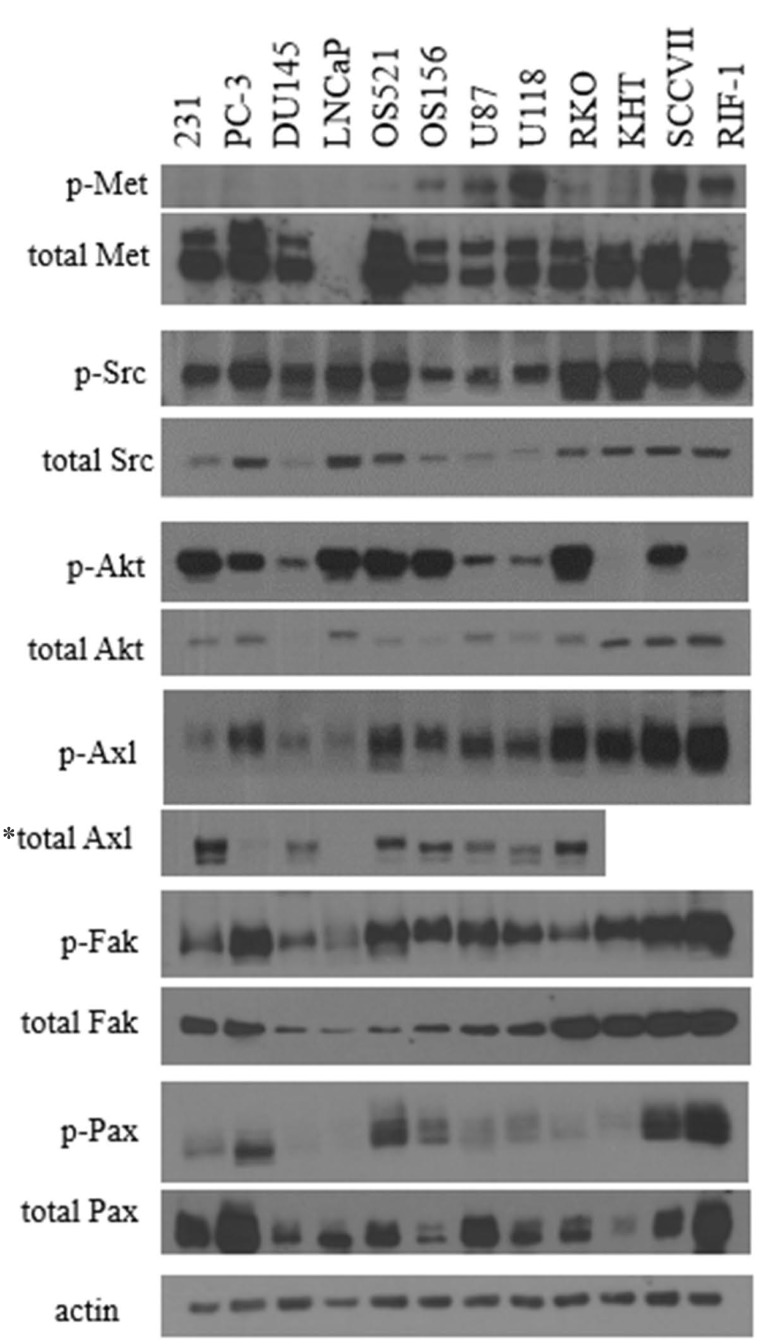
Comparison of phosphorylated and total protein between tumor cell lines Cell lysates were probed for phospho-EGFR (Y1068), total EGFR, phospho-Src (Y418), total Src, phospho-Akt (T308), total Akt, phospho-FAK (Y861), total FAK, phospho-Axl (Y776), total Axl, phospho-Pax (Y118), total Pax, phospho-Met (Y1234/1235), total Met, and actin. ^*^Total Axl antibody is specific for human.

Clinical trials of c-Met inhibitors typically select patients on the bases of tumor type, *MET* amplification, or c-Met protein overexpression. However, none of these surrogate markers have been shown to correlate with phosphorylation of c-Met, the indicator of pathway activity. In our studies, neither tumor type, gene expression, nor protein expression correlated with pathway activation. In fact, these surrogate markers have never been rigorously validated as reliable markers for c-Met activation, yet are routinely used in patient selection for clinical trials. Phosphorylation of c-Met and exon 14 deletion are the only indicators proven to correspond to pathway activity. Yet, c-Met is rarely mutated in the general cancer population, and phospho-Met has never been used as a selection criteria in clinical trials [24]. Unfortunately, this raises the question of whether lack of patient response to c-Met inhibition is an indication of an ineffective inhibitor, or the tumor lacking sufficient activation of the c-Met pathway. While our focus has been on the c-Met pathway, it is likely that the lack of pathway activation is an issue that involves other oncoproteins beyond c-Met. Many clinical trials utilize markers, including total protein, gene expression, as well as upstream or downstream proteins to select patients for clinical trials for a wide variety of targeted agents. In Figure 10, we observe the relationship between the activated protein state and total protein expression levels in a number of different proteins involved in cancer progression. Many of these proteins lack a correlation between total protein and the activation state. Therefore, it is not accurate to assume that expression of high levels of a protein implies activation of a pathway, and hence presence of an actionable target.

The critical evaluation of c-Met targeting agents is further complicated by the common use of high concentrations of exogenous HGF in the *in vitro* assessment of c-Met inhibitors. Exogenous HGF concentrations (typically 50 ng/mL, but as high as 10,000 ng/mL) which far exceed human serum levels (<1 ng/mL) are often added. While the addition of such high concentrations of HGF provides robust results, it is unlikely to be physiologically relevant.

In our studies, the human cancer cell lines DU145 and MDA-MB-231 showed a strong reduction of c-Met phosphorylation, migration, and invasion when treated with the c-Met inhibitor BMS-777607 in the presence of commonly used concentrations of HGF (25–50 ng/mL). However, when HGF was added at physiological concentrations (0.4–0.8 ng/mL), the cell lines failed to respond to the agent. Thus cell lines lacking response to c-Met inhibition could demonstrate sensitivity to c-Met inhibition in the presence of high exogenous levels of HGF. Our data suggest that concentrations of 25–50 ng/mL HGF strongly activate c-Met in paracrine-activated tumor cells. When c-Met activation results from these high exogenous HGF levels, it can be impaired by treatment with a c-Met inhibitor. In the presence of lower HGF concentrations, the cell lines do not respond to the inhibitor, likely because there is insufficient activation of c-Met. Conducting *in vitro* studies in such conditions may lead to an overestimation in the broad applicability of this type of inhibitor. While the authors urge greater diligence in assessment of these agents, our lab too has published c-Met inhibition studies utilizing extreme HGF concentrations [25].

While paracrine-activated tumor cells responded to c-Met inhibition only in the presence of very high concentrations of HGF, tumor cells that had a deletion of exon 14, as well as those that secreted HGF, thereby activating c-Met in an autocrine fashion, were intrinsically sensitive to c-Met inhibition. Both types of tumor cells showed sensitivity to c-Met inhibition in migration and invasion assays without the addition of any exogenous HGF. *In vivo* studies confirmed sensitivity to c-Met inhibition. In both angiogenesis and lung metastasis assays, these types of tumor cells were inhibited by treatment with the small molecule c-Met inhibitor BMS-777607.

Historically, *in vivo* preclinical investigations of c-Met inhibitors have been somewhat limited based on one report that murine HGF does not stimulate human c-Met [26]. In order to circumvent the issue of murine HGF being incompatible with human c-Met, genetically modified mouse models have been developed [27–29], however, some of these models also express higher levels of HGF than would be found in human serum, which as mentioned previously, may be problematic for realistic assessments of efficacy. Many reports have referenced the original study examining murine HGF and human c-Met, but to the best of our knowledge, no one has performed this experiment at the higher concentrations of HGF typically used *in vitro*. In Supplementary Figure 2, we compared the phosphorylation status of human c-Met when exposed to human or murine HGF at a range of concentrations, up to 50 ng/mL. Murine HGF did stimulate human c-Met, albeit at higher concentrations. While murine HGF resulted in substantially less phosphorylation than human HGF, it was interesting that 5 ng/mL murine HGF resulted in approximately as much phosphorylation as 0.5 ng/mL human HGF (a value realistic in humans). As seen in Supplementary Figure 3A, the NSG mouse model has HGF serum levels of approximately 5 ng/mL. As such, we chose to conduct *in vivo* studies of human tumor cell lines in the NSG mouse model. In intradermal angiogenesis and lung metastasis assays, the human tumor cell lines DU145 and MDA-MB-231 did not respond to c-Met inhibition. This is consistent with the lack of response seen in the *in vitro* studies conducted at 0.4–0.8 ng/mL HGF.

The concentration of HGF is clearly important in predicting response to c-Met inhibition in paracrine-activated tumor cells. This begs the question- what is the concentration of HGF within the tumor microenvironment? While many studies have quantified HGF in serum, as well as tumor homogenates (Supplementary Figure 3A, 3B), there have been no studies providing a volumetric assessment of HGF within a tumor. In order to make a more direct comparison between the concentrations used in *in vitro* studies and the tumor microenvironment, our lab utilized a microdialysis model to sample the concentration of HGF within the tumor of a live mouse. Our studies indicated that the concentration of HGF within a KHT tumor is approximately 0.06 ng/mL (Supplementary Figure 3C), which is roughly 100 fold lower than the serum concentrations of the host. In comparison, concentrations of HGF used in *in vitro* studies are typically 60–125 times the concentration found in human serum, and 5–10 times the concentration of HGF found in the serum of the C3H/HeJ mouse strain. While these findings are very preliminary, they do caution against assumptions of elevated intra-tumor HGF concentrations.

While gene amplification and total expression of c-Met have not shown strong correlations to phosphorylation of c-Met, phospho-Met remains the most accurate indication of pathway activity, and thus, sensitivity to c-Met inhibition. Although phospho-Met staining has proved difficult, it is possible and recently, a phospho-Met test has been made commercially available [30]. Advances in services such as this will hopefully eliminate the current reliance on sub-par surrogate markers.

There are limitations in this study. A significant factor that is oft-ignored in *in vitro* investigations involving c-Met and HGF is the presence of HGF activators in culture. While many cell lines may secrete HGF, it is secreted in an inactive, pro-form until cleaved by activators. Many cell lines also express HGF activators, but the status should be known when investigating autocrine-activated cell lines, as this will be an important factor in phosphorylation of Met. A second limitation of this study is the comparison of phosphorylation of Met via western blots. Unfortunately, this method may not allow the detection of minute changes in phosphorylation status. However, with the development of a commercially available phospho-Met assay, measuring activation status can become more precise. A third major limitation of this study is the promiscuity of the c-Met inhibitor BMS-777607. BMS-777607 also inhibits Ron, Axl, and Tyro3. As seen in Figure 10, the cell lines used in these studies also express Axl, and as such, the effects seen on metastasis suppression may be due, at least in part, to a suppression on the Axl, or potentially Ron/Tyro3 pathways. An additional study with a selective c-Met inhibitor would reveal important information, unfortunately, this study was not possible in our given time frame. The authors hope that future studies of autocrine and paracrine cell lines will include a more selective inhibitor.

To summarize, c-Met inhibitors have demonstrated disappointing efficacy in human trials. However, we believe c-Met inhibitors may have been inadequately evaluated due to (i) their assessment *in vitro* in the presence of abnormally high exogenous HGF concentrations, and (ii) their evaluation in patient populations without proven c-Met pathway activation. In tumors with demonstrable c-Met pathway activity, c-Met inhibitors may yet prove beneficial.

## MATERIALS AND METHODS

### Cell culture

See Supplementary Materials.

### Semi-quantitative real time PCR

Primers were designed as follows: human HGF, forward, 5ʹ-CTC ACA CCC GCT GGG AGT AC-3ʹ, reverse, 5ʹ-TCC TTG ACC TTG GAT GCA TTC-3ʹ; mouse HGF, forward, 5ʹ-GGC AAG GTG ACT TTG AAT GA-3ʹ, reverse, 5ʹ-CAC ATG GTC CTG ATC CAA TC-3ʹ; human c-Met, forward, 5ʹ-CTG CCT GCA ATC TAC AAG GT-3ʹ, reverse, 5ʹ-ATG GTC AGC CTT GTC CCT C-3ʹ; mouse c-Met, forward, 5ʹ-CCA GCA GCT TCA GTT ACC GG-3ʹ; human, GAPDH, forward 5ʹ-TGC ACC ACC AAC TGC TTA GC-3ʹ, reverse, 5ʹ-GGC ATG GAC TGT GGT CAT GAG-3ʹ; mouse GAPDH, forward, 5ʹ-GAG TTG TCA TAT TTC TCG T-3ʹ, reverse, 5ʹ-TAT GTC GTG GAG TCT ACT GGT-3ʹ. Cells were grown to 60–70% confluence. RNA was extracted using the Mini Prep kit (Qiagen). CDNA was prepared using the TaqMan Reverse kit (Applied Biosystems). All reactions were performed on the ABI7500 Fast Real-Time PCR.

System (Applied Biosystems). mRNA levels of tested genes were normalized to GAPDH according to the following formula: 2^(ΔΔCT)^, where CT is the threshold cycle.

### Western blot

Cells were harvested and disrupted in a radioimmunoprecipitation assay lysis buffer (50 mM Tris–HCl, pH 8.0, 150 mM NaCl, 0.1% SDS, 1% NP-40, 0.25% Sodium deoxycholate and 1 mM EDTA) with protease inhibitor cocktail, 1 mM NaF and 1 mM Na_3_VO_4_. Equal amounts (40 μg) of whole cell lysates were separated on an 8% SDS-PAGE gel. After electrophoresis, samples were electrotransferred to a nitrocellulose membrane (BioRad) and probed with the following primary antibodies at 4° C overnight: Phospho-c-Met (Y1234/Y1235) (Cell Signaling #3077), total Met (Cell Signaling #3127), and β-actin (Sigma #1978). After primary antibody incubation, the membranes were incubated with the species-appropriate horseradish peroxidase conjugated secondary antibody (Jackson ImmunoResearch), and detected with an enhanced chemiluminescence substrate (GE Healthcare and Perkin Elmer).

### ELISA

Cells were cultured in 60 mm dishes until approximately 60–70% confluency. 24 hours prior to harvest, media was discarded and replaced with fresh media. Conditioned media was collected and spun down to remove any floating cells, aliquoted and stored at –20° C until analysis with the appropriate ELISA kit (mouse/rat or human: R&D. Canine: Sigma). HGF is normalized to pg/10^6^ cells in figures.

### Exon 14 mutation detection

Primers for the murine tumor cell lines KHT and LLC were designed as follows: forward, 5ʹ- AGC CAG TAA TGA TCT CAA TG-3ʹ, reverse, 5ʹ-TCA GGA TAG GGG ACA GGT-3ʹ. Wild type cells were predicted to produce a transcript 570 base pairs in length, while those lacking exon 14 would produce a transcript 429 base pairs in length. Primers for the murine tumor cell lines B16F0, B16F1, B16F10, SCCVII and RIF-1 were designed as follows: forward, 5ʹ-GGT GCG GTC TCA ATA TCA GTA G-3ʹ, reverse, 5ʹ-CTT GGA CCA GCT CTG GAT TT-3ʹ. Wild type cells were predicted to produce a transcript 382 base pairs in length, while those lacking exon 14 would produce a transcript 241 base pairs in length. Transcripts were run on an agarose gel and visualized with ethidium bromide. Quick-Load 100 bp DNA ladder was used (New England Biolabs #N0551G).

### Reagents

BMS-777607 was kindly provided by Dr. Joseph Fargnoli (Bristol-Myer Squibb). For *in vitro* use, the powder was dissolved in dimethyl sulfoxide at a stock concentration of 10 mM, and stored in aliquots at –20° C until use. For *in vivo* use, BMS-777607 was dissolved in sterile-filtered 30% PEG 400 70% ddi H_2_O to a concentration of 3 mg/mL or 1.5 mg/mL, prepared daily before use, and administered via oral gavage at a volume of 0.01 mL/mg for a final dose of 30 mg/kg or 15 mg/mL. For *in vitro* use, recombinant human Hepatocyte Growth Factor (R&D Systems) was dissolved in sterile 0.1% BSA in PBS to a concentration of 50 μg/mL and aliquots were stored at –20° C until use.

### Cell migration

Cell migration was evaluated using 24-well 8.0 μm pore transwell chambers (BD Falcon). Cells were suspended in 200 μL of media and seeded into the upper chamber. 0.75 mL of media was placed into the lower chamber. BMS-777607 was added to both the upper and lower chambers. Cells were allowed to migrate for 24 hours at 37° C. After 24 hours, any cells remaining in the upper chamber were removed with a cotton swab, and the successfully migrated cells were stained with crystal violet and visually counted. DU145 cells were seeded at 2 × 10^4^ cells per insert. MDA-MB-231 cells were seeded at 2.5 × 10^4^ cells per insert. KHT cells were seeded at 2 × 10^3^ cells per insert. SCCVII cells were seeded at 5 × 10^2^ cells per insert.

### Intradermal angiogenesis assay

DU145 cells were injected intradermally into male nude mice (Envigo), and MDA-MB-231 cells were injected intradermally into female nude mice (Envigo). Cells were injected at four sites on the ventral side of the animal, at 10^5^ cells in 10 μL of PBS. Three mice were used per treatment group. Mice either received cells that were exposed to BMS-777607 for 24 hours prior to injection, or the mice received the drug orally every day for the duration of the experiment. Three days after tumor cell injection, mice were euthanized, skin flaps were removed, and the number of blood vessels growing into the tumor nodule was quantified visually on a stereoscope.

### Lung metastasis assay

Experimental metastasis was evaluated using a tail vein injection model. DU145 cells were injected at a concentration of 10^6^ cells in 200 μL of PBS into the tail vein of male NSG mice (bred in house), aged 2–6 months. MDA-MB-231-4715-LM2, a metastatic subline of MDA-MB-231, were used in this experiment as this cell line preferentially metastasizes to the lungs. The MDA-MB-231-4175-LM2 cell line responds to c-Met inhibition in a similar manner as its parental line (Supplementary Figure 1). MDA-MB-231-4715-LM2 cells were injected at a concentration of 2 × 10^3^ cells in 200 μL of PBS into the tail vein of female NSG mice (bred in house), aged 2–6 months. Mice received BMS-777607 via oral gavage at a concentration of 30 mg/kg daily, for five days after tumor cell injection. Both DU145 and MDA-MB-231-4715-LM2 lung nodules were allowed to grow for 7 weeks. After the incubation period, mice were euthanized, lungs were placed in Bouin's solution for 24 hours, then transferred to isopropyl alcohol. Lung nodules were visually counted using a dissecting microscope. For the MDA-MB-231-4175-LM2 model, this experiment was repeated twice. As there was a fair amount of variability not seen in other cell lines, this experiment was repeated to confirm our findings.

### Statistical analysis

Multiple comparisons ANOVA was employed for *in vitro* data analysis, and a Mann-Whitney U test was employed for *in vivo* data analysis by GraphPad Prism 6.0. A threshold of p < 0.05 was defined as statistically significant.

## SUPPLEMENTARY MATERIALS FIGURES AND TABLES


