# Supplementary material for: Failures in preclinical and clinical trials of c-Met inhibitors: evaluation of pathway activity as a promising selection criterion
Source: Oncotarget. 2019 Jan 4;10(2):184–97. doi: 10.18632/oncotarget.26546 (PMC6349429; doi:10.18632/oncotarget.26546)
Supplement: Supplementary file 1 [file oncotarget-10-184-s001.pdf]

Failures in preclinical and clinical trials of c-Met inhibitors: evaluation of pathway activity as a promising selection criterion

SUPPLEMENTARY MATERIALS

Cell Culture

MDA-MB-231 is a human breast carcinoma cell line acquired from ATCC, cultured in Dulbecco's MEM (DMEM). PC-3 is a human prostate carcinoma cell line acquired from ATCC, cultured in Ham's media. DU145 is a human prostate carcinoma cell line acquired from ATCC, cultured in Eagle's Minimum Essential Media (MEM). LNCaP is a human prostate carcinoma cell line acquired from ATCC, and is cultured in RPMI-1640 media. OS156 is a human osteosarcoma cell lines, acquired from Parker Gibbs of the University of Florida, and cultured in Ham's media. OS521 is a human osteosarcoma cell lines, acquired from Parker Gibbs of the University of Florida, and cultured in a 1:1 mixture of DMEM and Ham's. U87 is a human glioblastoma cell line acquired from John Buatti of the University of Iowa, and is cultured in Eagle's MEM.

U118 is a human glioblastoma cell lines acquired from John Buatti of the University of Iowa, and is cultured in DMEM.

RKO is a human colorectal carcinoma acquired from ATCC, and is cultured in Eagle's MEM. KHT is a murine fibrosarcoma cell line acquired from Robert Kallman of Stanford University, and is cultured in alpha MEM. SCCVII is a murine fibrosarcoma cell line acquired from Herman Suit of Boston University, and is cultured in alpha MEM. RIF-1 is a murine fibrosarcoma cell line acquired from P Twentyman of Stanford University, and is cultured in alpha MEM.

Additional cell lines: L0, L1, L2 (human glioblastoma), MG-63 (human osteosarcoma) Malme-3, Malme-3M, WI-38 (human fibroblast), HL-60 (human leukemia), Caki-1, Caki-2 (human renal cell carcinoma), H1993 (human lung carcinoma), UMSCC1 (human head and neck squamous cell carcinoma), SK-OV-3, OW1, SKA, MLS (human ovarian carcinoma), A549 (human lung carcinoma), LoVo, HCT116, Caco-2, HT29 (human colorectal), 293 (human embryonic kidney cells), EMT6 (murine mammary carcinoma), B16F0, B16F1, B16F10 (murine melanoma), LLC (murine Lewis lung carcinoma), M109, 3T3 (human fibroblast), RAW (human macrophages), D1K2-T1, D1K2-T3, MTAMF control/hHGF (murine tumor associated fibroblast), POS, HMPOS (canine osteosarcoma).

All media was supplemented with 10% fetal bovine serum (Gibco) and 1% Pen-Strep (Gibco). All cell lines are maintained in a humidified 5% CO2 chamber at 37° C.

Western blot

Cells were harvested and disrupted in a radioimmunoprecipitation assay lysis buffer (50 mM Tris–HCl, pH 8.0, 150 mM NaCl, 0.1% SDS, 1% NP-40, 0.25% Sodium deoxycholate and 1 mM EDTA) with protease inhibitor cocktail, 1 mM NaF and 1 mM Na_3VO_4. Equal amounts (40 µg) of whole cell lysates were separated on an 8% SDS-PAGE gel. After electrophoresis, samples were electrotransferred to a nitrocellulose membrane (BioRad) and probed with the following primary antibodies at 4°C overnight: phospho-EGFR (Y1068) (Cell Signaling #2236), total EGFR (Cell Signaling #4267), phospho-Src (Y416) (Cell Signaling #2101), total Src (Cell Signaling #2109), phospho-Akt (S473) (Cell Signaling #9271), total Akt (Cell Signaling #9272), phospho-Fak (Y861) (Invitrogen #44-626G), total Fak (Cell Signaling #3285), phospho-Axl (R&D Systems #AF2228), total Axl (Cell Signaling #8661), phospho-Pax (Y118) (Cell Signaling #2541), total Pax (Cell Signaling #2542), phospho-c-Met (Y1234/Y1235) (Cell Signaling #3077), total Met (Cell Signaling #3127), and β-actin (Sigma #1978). After primary antibody incubation, the membranes were incubated with the species-appropriate horseradish peroxidase conjugated secondary antibody (Jackson ImmunoResearch), and detected with an enhanced chemiluminescence substrate (GE Healthcare and Perkin Elmer).

Tumor homogenates

Six to eight week old female C3H/HeJ mice (Jackson) were injected intramuscularly with 10^5 KHT murine fibrosarcoma cells. Beginning 5 days after injection, leg-bearing tumors were measured daily using a ruler with punched with holes of a specified size. Mice were euthanized when tumors reached a size of 150–950 mm^3. Tumors were rinsed in PBS, weighed, and minced into 1–2 mm pieces. Tumors were placed in a tube with a small volume of PBS and homogenized. After homogenization, an equal volume of Cell Lysis Buffer 2

(R&D) was added, for a final volume of PBS/buffer at 4–8 times the volume of the tissue. After the addition of lysis buffer, the sample was allowed to incubate for 30 minutes at room temperature on a rocker. The sample was centrifuged at 12,000 rpm for 10 minutes at 4° C and the aqueous layer was removed, aliquoted, and stored at –20° C until analysis by ELISA (R&D).

Microdialysis

Five to six week old female C3H/HeJ mice (Jackson) were injected with 2×10^4 or 2×10^5

KHT fibrosarcoma tumor cells intramuscularly into the left hind leg. Tumors were measured daily till reaching a volume of 1000 mm^3. Microdialysis probes were primed with T1 buffer (CMA) at 2 µL/minute prior to insertion. Anesthesia was induced using 3% isoflurane and maintained using 1.5% isoflurane. A single probe was inserted into the center of the tumor, and a second probe was inserted into the healthy muscle of the opposite leg with the use of splittable guide cannulas. The probes were allowed to rest in the tissue for 30 minutes, then flushed at a flow rate of 1 µL/minute using an infusion pump and a peristaltic pump (Harvard Apparatus). 120 µL of dialysate was collected at a flow rate of 1 µL/minute, and frozen at –20° C until analysis by ELISA (R&D). The concentration of HGF was calculated using the value determined in the retrodialysis calibration.

Retrodialysis calibration

Microdialysis probes (CMA) were used to sample HGF concentrations within a tumor of a live mouse. Microdialysis probes are composed of a membrane designed to allow the exchange of solutes to permit sampling of proteins in the extracellular space within a tumor microenvironment. Relative recovery approaches 100% as the flow rate approaches zero. The flow rate of 1 µL/minute was determined to be the fastest flow rate that provided a resulting dialysate protein concentration well within the assay sensitivity. Probes were calibrated for HGF recovery using the following *in vivo* retrodialysis equation:

$$R = (C_{perfusate} - C_{dialysate}) = C_{perfusate}.$$

Five to six week old female C3H/HeJ mice (Jackson) were injected with 2×10^5 KHT fibrosarcoma tumor cells intramuscularly into the left hind leg, and allowed to grow to a size of 1000 mm^3. Various quantities of HGF were diluted in T1 buffer (CMA) for use as the perfusate. Probes were primed at a flow rate of 2 µL/minute prior to insertion. Anesthesia was induced using 3% isoflurane and maintained using 1.5% isoflurane. The probe was inserted into the tumor with a splittable guide cannula. After probe insertion, the tissue was allowed to rest for 30 minutes, then flushed at a flow rate of 1 µL/minute using an infusion pump and a peristaltic pump (Harvard Apparatus). The resulting dialysate, collected at a flow rate of 1 µL/minute, was analyzed via ELISA (R&D) for retrodialysis calculation. The resulting value of 13.12% was used to determine relative recovery of HGF.

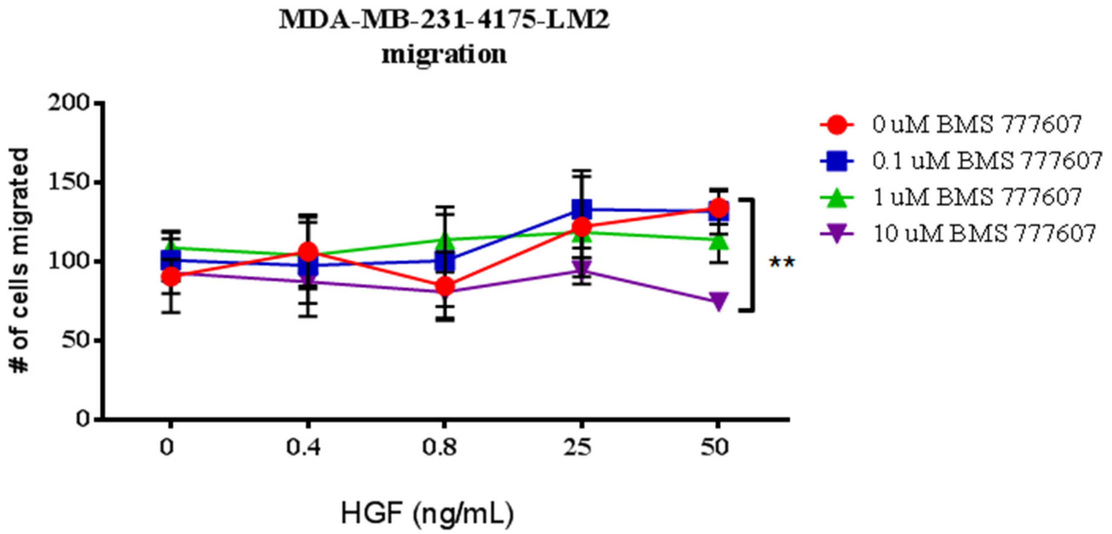

Supplementary Figure 1: Transwell migration assay of MDA-MB-231-4175-LM2 tumor cells. Cells were treated with BMS-777607 and/or HGF and allowed to migrate for 24 hours through an 8 um pore membrane. Results are a combination of three independent experiments. Multiple comparisons ANOVA was performed using GraphPad Prism 6.0, with $p < 0.05$ considered significant. Mean with SEM bars are shown.

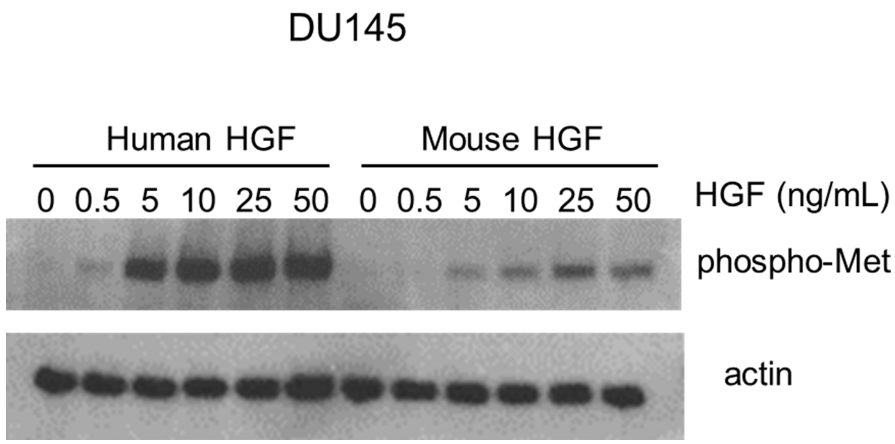

Supplementary Figure 2: Phosphorylation of c-Met after 15 minute treatment with human and mouse HGF in the DU145 cell line. Cell lysates were probed for phospho-Met (Y1234/1235) and actin.

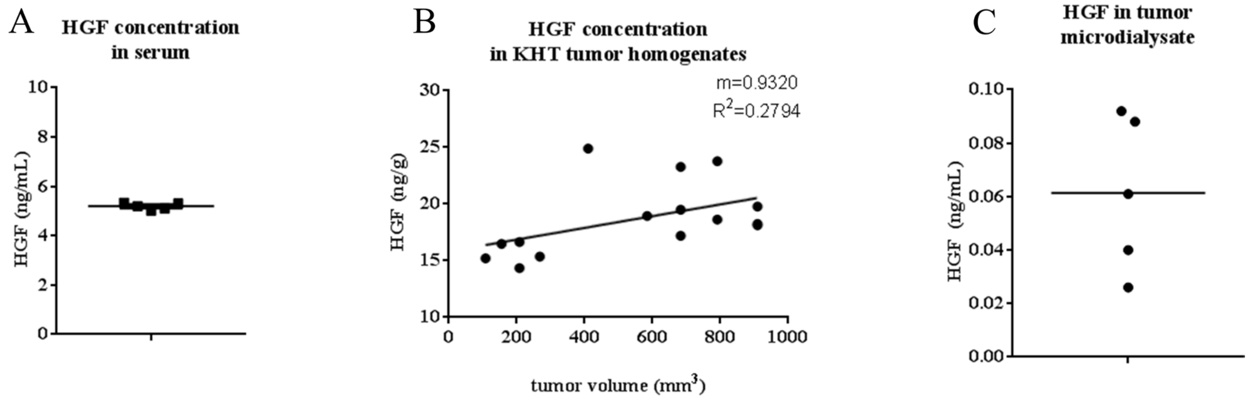

Supplementary Figure 3: Characterization of HGF *in vivo*. (**A**) HGF concentrations within serum of C3H/HeJ mice. (**B**) Concentration of HGF in KHT tumor homogenates harvested from C3H/HeJ mice. (**C**) Measurement of HGF within a KHT tumor grown in a C3H/HeJ mouse sampled via microdialysis. ELISA was used to quantify HGF.
